# Albuminuria in Health

**Published:** 1887-09

**Authors:** 


### *ALBUMINURIA IN HEALTH.*

The occasional appearance of albumen in the urine of apparently healthy persons is a fact of no mean clinical importance. The British practitioner is quite aware of the usually grave signification of albuminuria, even when the albumen is scanty, and he is perfectly cognizant of the ordinary chemical test for that compound. Hence, it is important that he, as well as the hospital physician, should not jump to the conclusion that a trace of albumen necessarily means serious kidney disease. Dr. C. von Noorden, of Giessen, has recently contributed a monograph on "Albuminuria in Healthy Persons" to the *Deutsche Archiv. für Klinische Medicin.* He classes "physiological" albuminuria into three groups. In the first group, the albuminuria is generally found in weakly youths between the ages of puberty and twenty, rarely in children or in adults. The presence of albumen is discovered in these cases either during clinical statistical researches, or else in persons who send for the medical attendant because they feel faint, weak, or otherwise slightly indisposed. The proportion of albumen differs greatly at intervals of a few hours. It may run up from 0.0 to 0.5 per cent., or higher, in a single morning. Rarely, if ever, is the urine continuously albuminous all day. These conditions are very characteristic of physiological albuminuria, and do not exist in any form of nephritis. The urine is pale, clear, and generally, but not always, of high specific gravity. The albumen is always coagulable on boiling. Occasionally a globulin-like compound has been detected in excess of the serum-albumen. Casts very rarely are found, and, if present, they are hyaline, never epithelial. The albumen is always to be found in greatest quantity before noon. In some cases of physiological albuminuria no abnormal general condition could be found; in others, muscular pains, errors of diet or mental excitement have been observed and assigned as causes of this condition. No evidence of renal disease has ever been proved, nor of altered conditions of the blood. Dr. von Noorden believes that it is more likely due to blood-changes themselves, possibly caused by slight renal disease, than by disturbed filtration in the tubuli uriniferi, as Leube has suggested. In the second class of cases, mucin is present as well as albumen. In this case, again, the albuminuria is most marked before noon. The mucin might be derived from the lower part of the urinary tract, or from the kidney itself. The proportion of albumen is very variable, and much influenced by bodily exertion. In raw recruits it is most abundant after heavy drill. Dr. von Noorden believes that this class represents mild vesical catarrh. The third class of cases, on the other hand, appears to represent slight renal catarrh, insufficient to cause the general and local symptoms of renal disease, just as, in the second, the subjective signs of cystitis are present. In striking contrast to the first class, the albuminuria may last for a whole day and then disappear, or may be found only before noon, yet in regular but very small proportions. No mucin can be detected, but hyaline, and sometimes epithelial casts, and even red corpuscles, are generally present. The first class is evidently the purest kind of "physiological" albuminuria. Yet this term is still questionable, for a trifling amount of disease in the genito-urinary apparatus is a more probable cause of the condition in question than any unusual "physiological" tissue-change, caused by exertions after heavy meals, etc. Physiological albuminuria, then, must be held to imply albuminuria in persons who appear to be otherwise healthy, though local disease is, in all probability, present to an extent insufficient to produce any other symptom.—*British Medical Journal.*

